# Regional Differences in Awareness of Oral Frailty and Associated Individual and Municipal Factors: A Cross-Sectional Study

**DOI:** 10.3390/healthcare13151916

**Published:** 2025-08-05

**Authors:** Nandin Uchral Altanbagana, Koichiro Irie, Wenqun Song, Shinya Fuchida, Jun Aida, Tatsuo Yamamoto

**Affiliations:** 1 Department of Preventive Dentistry and Dental Public Health, Kanagawa Dental University, 82 Inaoka-cho, Yokosuka 238-8580, Kanagawa, Japan; nandin@kdu.ac.jp (N.U.A.); song@kdu.ac.jp (W.S.); 2 Department of Oral Health, Medical and Dental Sciences, Graduate School of Biomedical Sciences, Nagasaki University, 1-12-4 Sakamoto, Nagasaki 852-8523, Nagasaki, Japan; iriko@nagasaki-u.ac.jp; 3 Department of Education Planning, Kanagawa Dental University, 82 Inaoka-cho, Yokosuka 238-8580, Kanagawa, Japan; fuchida@kdu.ac.jp; 4 Department of Dental Public Health, Graduate School of Medical and Dental Sciences, Institute of Science Tokyo, 1-5-45 Yushima, Bunkyo-ku, Tokyo 113-8510, Japan; aida.ohp@tmd.ac.jp

**Keywords:** oral frailty, awareness, regional difference, individual factor, regional factor, cross-sectional study, multilevel Poisson regression model

## Abstract

**Background/Objectives**: Despite growing interest in oral frailty as a public health issue, no nationwide study has assessed regional differences in oral frailty awareness, and the factors associated with such differences remain unclear. This study investigated regional differences in oral frailty awareness among older adults in Japan and identified the associated individual- and municipal-level factors, focusing on local policy measures and community-based oral health programs. **Methods**: A cross-sectional analysis was conducted using data from the 2022 wave of the Japan Gerontological Evaluation Study. The analytical sample comprised 20,330 community-dwelling adults aged ≥65 years from 66 municipalities. Awareness of oral frailty was assessed via self-administered questionnaires. Individual- and municipal-level variables were analyzed using multilevel Poisson regression models to calculate prevalence ratios (PRs). **Results**: Awareness of oral frailty varied widely across municipalities, ranging from 15.3% to 47.1%. Multilevel analysis showed that being male (PR: 1.10), having ≤9 years (PR: 1.10) or 10 to 12 years of education (PR: 1.04), having oral frailty (PR: 1.04), and lacking civic participation (PR: 1.06) were significantly associated with lack of awareness. No significant associations were found with municipal-level variables such as dental health ordinances, volunteer training programs, or population density. **Conclusions**: The study found substantial regional variation in oral frailty awareness. However, this variation was explained primarily by individual-level characteristics. Public health strategies should focus on enhancing awareness among socially vulnerable groups—especially men, individuals with low educational attainment, and those not engaged in civic activities—through targeted interventions and community-based initiatives.

## 1. Introduction

Frailty has emerged as an increasingly important public health issue amid global population aging, including in Japan [1]. More recently, oral frailty has been recognized as a pivotal aspect of overall frailty, with prior studies highlighting associations between oral health status and general frailty among older adults [2,3]. The Japan Dental Association defines oral frailty as a progressive condition involving age-related deterioration in oral health, such as tooth loss, poor oral hygiene, and a decline in oral function, combined with decreased interest in oral care and diminished physical and mental resilience [4]. This condition may lead to eating dysfunction, and a consequent further deterioration of both physical and cognitive capacities. Oral frailty has been associated with a range of adverse outcomes, including poor nutritional status [5], sarcopenia [6], increased falling risk [7], depression [8], cognitive decline [9], and reduced civic participation [10], highlighting its broad impact on older adults’ health and well-being.

Awareness of oral frailty plays a critical role in promoting preventive behaviors. Higher levels of awareness have been shown to encourage proactive health practices, including better oral hygiene and regular dental care, thereby reducing the risk of oral diseases [11,12]. Accordingly, the Japan Dental Association has set a 50% awareness of oral frailty by 2025 as a national target [13]. However, a recent prefectural survey reported an awareness rate of only 29.6%, indicating a substantial gap [14]. Moreover, awareness tends to be lower among socially vulnerable groups such as older adults, individuals at risk of oral frailty, and those requiring home dental care [14]. Recent studies have also shown that strong civic engagement and participation in community activities are associated with higher levels of awareness and a reduced risk of oral frailty [15]. These findings suggest that both individual characteristics and community-level social capital may play a crucial role in fostering awareness and encouraging preventive action.

In response to the growing need for prevention, municipalities in Japan have implemented various oral frailty initiatives in collaboration with dental associations, dental hygienist organizations, and other stakeholders. These include community-based education, oral health screening, individualized guidance, the distribution of informational materials, and public lectures [16,17]. Some municipalities have enacted dental health ordinances that address oral frailty explicitly. While these efforts are expected to raise awareness, few studies have systematically examined their impact. Assessing the effectiveness of such measures requires a consideration of both individual- and community-level factors, including how local policy frameworks and social infrastructure contribute to awareness and behavioral change.

The aim of this study was to investigate regional differences in oral frailty awareness among older adults in Japan and to identify the associated individual- and municipal-level factors, with a focus on local policy measures and community-based oral health programs.

## 2. Materials and Methods

### 2.1. Study Design and Participants

**Figure 1:**
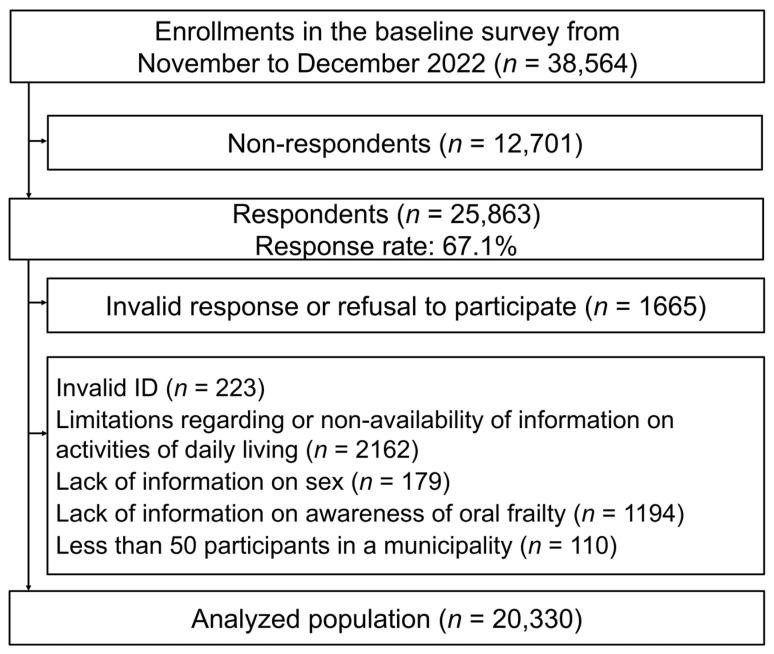
Flow chart of participant selection process.

This study utilized cross-sectional data taken from the 2022 wave of the Japan Gerontological Evaluation Study (JAGES), a nationwide epidemiological study on the social determinants of healthy aging among nondisabled individuals aged ≥65 years. In 2022, the JAGES survey targeted 338,742 community-dwelling older adults from 75 municipalities in 24 prefectures across Japan, of whom 228,119 responded. A subsample-specific version of the self-administered questionnaire (Version D), which included items on oral frailty awareness, was distributed to 38,564 randomly selected individuals residing in 69 municipalities. Of these, 25,863 individuals returned completed questionnaires (response rate: 67.1%; Figure 1).

To these respondents, several exclusion criteria were applied: individuals who refused to participate or who submitted invalid responses (*n* = 1665), invalid respondent IDs (*n* = 223), no or insufficient information regarding independence in daily living (*n* = 2162), no data on sex (*n* = 179), or no responses related to awareness of oral frailty (*n* = 1194). In addition, participants residing in municipalities with fewer than 50 respondents were excluded (*n* = 110) to ensure the stability of the municipal-level estimates. After these exclusions, 20,330 participants from 66 municipalities were included in the analytical sample used for this study.

### 2.2. Variables

#### 2.2.1. Dependent Variable

The dependent variable was awareness of oral frailty. This was assessed using the question, “Are you familiar with the term ‘oral frailty’?” Responses of “very familiar” and “have heard of the term” were classified as “aware,” while the response of “not familiar” was classified as “not aware.”

#### 2.2.2. Individual-Level Independent Variables

Individual-level variables were obtained from a self-administered questionnaire; these included sex, age group, educational attainment, equivalent income, current illness, oral frailty, drinking habits, smoking habits, frequency of going out, average daily walking time, frequency of meeting friends, civic participation, reciprocity, social cohesion, family structure, and housing type. Educational attainment was categorized as “≤9 years,” “10−12 years,” “≥13 years,” and “unknown” (i.e., other responses or missing data). Equivalent income was calculated by dividing household income by the square root of the number of household members to adjust for household size. Presence of current illness was based on a self-reported diagnosis of any of the following: hypertension; stroke; heart disease; diabetes; hyperlipidemia; respiratory disease; gastrointestinal, liver, or gallbladder disease; kidney or prostate disease; musculoskeletal disease; trauma; cancer; hematological or immune disorders; depression; dementia; Parkinson’s disease; or visual/hearing impairment. Responses were dichotomized as “yes” or “no.”

Civic participation, reciprocity, and social cohesion were assessed using methods described by the previous study [18]. Social cohesion was measured using three items: (1) “Do you think individuals in your community are generally trustworthy?” (2) “Do you think individuals in your community often try to be helpful to others?” and (3) “Do you have an attachment to your local area?” Participants who answered “yes” to at least one of the three questions were categorized as having social cohesion, and the rest were categorized as not having it. Civic participation was assessed using the question, “How often do you participate in a volunteer group, a sports group, or a hobby activity?” Participants who reported participating in any of the three group types at least once a month (from “1−3 times a month” to “≥4 times a week”) were categorized as “yes,” and those who responded “a few times a year” or “never” were categorized as “no.” Reciprocity was assessed using three items: (1) “Do you have someone who listens to your concerns and complaints?” (emotional support received), (2) “Do you listen to someone else’s concerns and complaints?” (emotional support provided), and (3) “Do you have someone who looks after you when you are sick?” (instrumental support received). Participants who answered “yes” to at least one item were categorized as having reciprocity; those who answered “no” to all items were categorized as not having reciprocity.

Drinking habits were categorized as “current” (currently drinking), “past” (quit within the past ≥5 years), “non-drinker” (never drank), and “unknown” (non-response). Smoking habits were classified similarly: “current,” “past,” “non-smoker,” and “unknown.”

Frequency of going out was grouped into five categories: “≥5 times a week,” “4 times a week,” “2−3 times a week,” “once a week,” and “not going out” (which combined “1−3 times a month” or “a few times a year”). Non-responses were categorized as “unknown.” Average daily walking time was classified as “<30 min,” “30−59 min,” and “≥60 min” (combining “60−89 min” and “≥90 min”); non-responses were coded as “unknown.”

Oral frailty was assessed using a predictive model [19], which incorporates age, number of teeth, difficulty eating tough foods, and choking. The number of teeth was self-reported based on several categories (≥20, 10−19, 1−9, 0) and dichotomized as ≥20 or ≤19. Difficulty eating tough foods was assessed with the question, “Do you have any difficulties eating tough foods now compared with 6 months previously?” Choking was assessed using the question, “Have you recently choked on your tea or soup?” Both were dichotomized into “yes” or “no” responses, based on the Basic Checklist used in Japan’s long-term care insurance system [20].

#### 2.2.3. Municipal-Level Independent Variable

This study obtained information on municipal oral frailty prevention programs via a postal questionnaire survey conducted between 20 December 2022 and 31 January 2023, targeting 66 municipalities. Responses were received from 48 municipalities, yielding a response rate of 72.7%. For non-responding municipalities, the relevant information was collected from official municipal websites. The survey collected information on the respondent’s name, contact information, and job title, as well as the presence or absence of municipal dental health ordinances and (if present) whether they included measures explicitly related to oral frailty. The same questions were asked regarding prefectural dental health ordinances. In addition, the survey inquired about the existence of oral frailty prevention initiatives implemented before fiscal year 2022, such as leaflet distribution, community lectures, and volunteer training programs for residents.

Information on population and municipal habitable land area (in hectares) was obtained from the 2020 Social and Demographic Statistics [21]. Population density (persons/km^2^) was calculated by dividing the total population by the municipal area, with hectares converted to square kilometers. Based on population density, municipalities were classified into four categories: metropolitan area (≥4000 persons/km^2^), urban area (2000−3999 persons/km^2^), semi-urban area (200−1999 persons/km^2^), and rural area (<200 persons/km^2^) [22].

Municipal-level social capital was calculated as the mean of aggregated individual-level binary variables (coded as 0 or 1) for civic participation, reciprocity, and social cohesion within each municipality.

### 2.3. Statistical Analyses

At the individual level, associations between awareness of oral frailty and each covariate were assessed using the chi-square test. At the municipal level, associations between the municipal-level variables and the proportion of individuals who were aware of oral frailty were examined using the Mann–Whitney U test or Kruskal–Wallis test. A two-level multilevel Poisson regression analysis was conducted, with lack of oral frailty awareness as the dependent variable (coded as 1 for “not aware” and 0 for “aware”). For Model 1, each individual- and municipal-level variable was entered into the model separately. For the municipal-level analysis, variables with a *p*-value of <0.20 in the univariate analysis were included. Model 2 included only sex and age. Model 3 included all individual- and municipal-level variables.

As a sensitivity analysis, cross-tabulations and two-level multilevel Poisson regression analyses were conducted using an alternative definition of the dependent variable. In this analysis, only the “very familiar” responses were categorized as “aware,” while “have heard of the term” and “not familiar” were combined and categorized as “not aware.” This approach was based on the assumption that individuals who have merely “heard of the term” are less likely to engage in health-promoting behaviors than are those who are “very familiar” with the concept of oral frailty. Furthermore, higher oral health literacy has been associated with more frequent preventive dental visits and better oral health outcomes [23].

All statistical analyses, except the multilevel analyses, were performed using IBM SPSS Statistics version 28.0 (SPSS Japan, Inc., Tokyo, Japan). The multilevel analyses were conducted using MLwiN version 3.10 (Centre for Multilevel Modelling, University of Bristol, UK). A two-sided significance level of 5% was used for all tests.

### 2.4. Ethical Issues

The 2022 JAGES survey was approved by the Ethics Committee on Research of Human Subjects at the Chiba University Graduate School of Medicine (approval no. M10460) and the Ethics Committee of Kanagawa Dental University (approval no. 988). Informed consent was obtained from all participants. This study was reported in accordance with the Strengthening the Reporting of Observational Studies in Epidemiology guidelines.

## 3. Results

**Figure 2:**
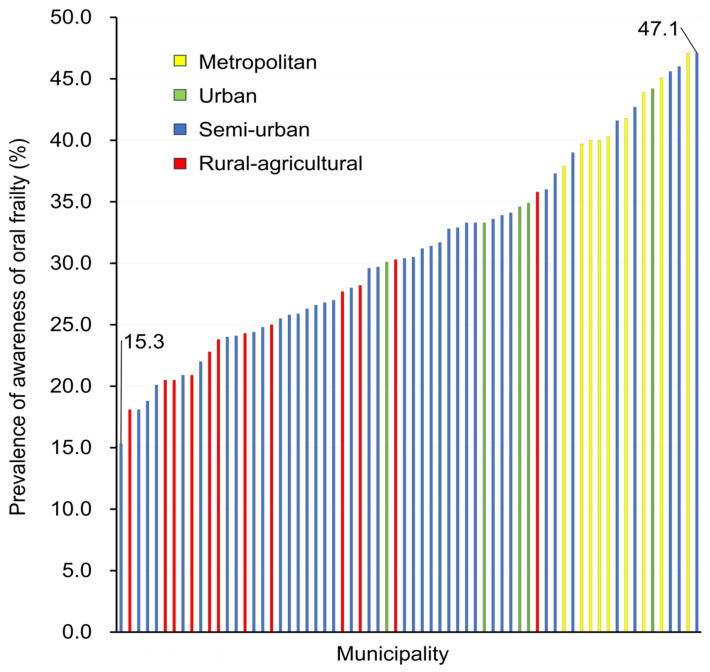
Prevalence of awareness of oral frailty across 66 municipalities.

Figure 2 presents the awareness levels concerning oral frailty across the 66 municipalities, which ranged from 15.3% to 47.1%, with a median of 30.4%. Awareness was generally lower in rural and agricultural areas and higher in metropolitan areas.

Table 1 presents the associations between awareness of oral frailty and individual-level variables. Significant associations were observed for all variables (*p* < 0.001). Individuals who were not aware of oral frailty were more likely to be male and older; have lower educational attainment and income; report no current illness; exhibit signs of oral frailty; have a history of alcohol consumption; currently smoke; go out infrequently; walk less per day on average; meet friends less frequently; and lack civic participation, reciprocity, and social cohesion. They were also more likely to live in two-person households and rental housing.

Table 2 presents the associations between awareness levels concerning oral frailty and municipal-level variables. The variables that showed a statistically significant association (*p* < 0.20) included explicit oral frailty measures in prefectural dental health ordinances, the presence of volunteer training programs for residents as part of oral frailty-related initiatives, and population density.

Table 3 displays the results of the multilevel Poisson regression analysis. In Model 1, the following variables were significantly associated with lack of awareness of oral frailty: being male; age ≥85 years (reference: 65−69 years); educational attainment of ≤9 years or 10−12 years (reference: ≥13 years); equivalent income ≤JPY 1.99 million (reference: ≥JPY 4 million); presence of oral frailty; current or past alcohol consumption (reference: none); current or past smoking (reference: none); not going out at all (reference: ≥5 times per week); average daily walking time of <30 min (reference: ≥60 min); infrequent contact with friends (reference: at least once a week); lack of civic participation; lack of social cohesion; housing type—owner-occupied (single-family) or rented (reference: owner-occupied, multi-family); and residence in semi-urban or rural areas (reference: metropolitan area). The municipal-level variance in the null model was 0.0008 (standard error = 0.0005). In Model 2, which included only sex and age, both variables were significantly associated with lack of awareness of oral frailty. In Model 3, which included all individual- and municipal-level variables, the following remained significantly associated with lower oral frailty awareness: male sex, educational attainment of ≤9 years or 10−12 years (reference: ≥13 years), presence of oral frailty, and lack of civic participation.

The results of the multilevel Poisson regression analysis from the sensitivity analysis (Table 4) indicated that lack of awareness of oral frailty was significantly associated with the following characteristics: male sex; educational attainment of ≤9 years or 10−12 years (reference: ≥13 years); equivalent income of ≤JPY 1.99 million (reference: ≥JPY 4 million); and lack of civic participation.

## 4. Discussion

This study is the first to investigate regional differences in awareness of oral frailty across Japan and to examine the associated individual- and municipal-level factors using multilevel analysis. The results revealed a substantial variation in awareness, ranging from 15% to 47%, a more than threefold difference across municipalities. The municipalities included in this study were those that responded to an invitation from researchers to engage in collaborative research efforts, including regional assessments through inter-municipality comparisons. As participation was limited to municipalities with a demonstrated enthusiasm for joint research, the disparities observed in inter-municipality comparisons may be underestimated. Consequently, the generalizability of these findings to municipalities with fewer officials committed to research and evidence-based policymaking may be constrained. These considerations highlight the necessity for tailored strategies to enhance awareness of oral frailty, with careful attention to regional contexts and inequities.

The results of the multilevel analysis (Model 3) showed that, at the individual level, persons who were men, had lower educational attainment, exhibited oral frailty, and lacked civic participation were independently associated with lack of awareness of oral frailty. At the municipal level, regional factors such as the presence of prefectural ordinances, oral frailty prevention programs, social capital, and population density were not significantly associated with awareness. Furthermore, the municipal-level variance, which was already low (0.0008) in the null model, decreased slightly from 0.0009 in Model 2 (adjusted for sex and age) to 0.0000 in Model 3, which included both individual- and municipal-level variables. These findings carry significant implications for public health practice. They suggest that regional disparities in oral frailty awareness are predominantly driven by individual-level characteristics within communities, highlighting the need for targeted interventions tailored to these factors. Moreover, this study extends the current literature by demonstrating, for the first time in a nationwide sample, how municipal-level policies and programs may interact with individual characteristics to influence oral frailty awareness. This contributes important insights into the design of community-based oral health strategies.

The results of this study showed that men were less aware of oral frailty than women. This association between sex and awareness is consistent with the findings of a study conducted in one prefecture of Japan [14]. Previous research has also indicated that men are more likely than women to experience oral frailty [15], possess less oral health knowledge, engage less frequently in appropriate oral health behaviors [24], and exhibit lower levels of individual social capital [25]. These findings suggest that men may represent a particularly important target population for interventions aimed at improving awareness of oral frailty.

The age variable was significantly associated with awareness in Models 1 and 2 but not in Model 3. This finding may have occurred because age is incorporated into the estimation of oral frailty status. Indeed, when the oral frailty variable was excluded from Model 3, age became significantly associated with awareness (data not shown).

Regarding educational attainment, individuals with fewer years of education were less likely to be aware of oral frailty. This finding is consistent with previous reports indicating that educational attainment is positively associated with general oral health knowledge [26,27]. Other studies have shown that individuals with lower educational attainment are at increased risk of oral frailty [15,28]. Furthermore, the study revealed that limited oral health knowledge and unfavorable attitudes among older adults, particularly those with lower educational attainment, were associated with suboptimal oral health behaviors and an increased risk of frailty [29]. These findings highlight the need for targeted strategies designed to raise awareness of oral frailty among individuals with lower levels of educational attainment.

This study also found that individuals with oral frailty were less likely to be aware of their condition than those without. Similarly, the previous study reported a significant association between oral frailty risk and lack of awareness levels, consistent with our findings [14]. These results suggest that individuals affected by oral frailty may be insufficiently cognizant of the condition and its associated risks. Therefore, enhancing awareness among those already experiencing oral frailty is essential for its prevention and early detection.

Among the individual-level aspects of social capital, lower civic participation was associated with lack of awareness of oral frailty. Although causality cannot be inferred due to the cross-sectional design of this study, initiatives that promote civic participation may help improve awareness.

We hypothesized that awareness of oral frailty would be higher in areas with high social capital. While civic participation at the individual level was significantly associated with awareness, no such association was observed for social capital at the municipal level. Previous studies have shown that living in communities with high civic participation levels is associated with a higher risk of oral frailty [15,30], a higher number of teeth [31], and a lower risk of tooth loss [32]. Notably, these studies constructed social capital variables at the school district level. By contrast, this study assessed social capital at the municipal level because its participant sample was relatively small. The choice of geographical units used to measure social capital may have influenced the findings. Future research should use larger sample sizes and more granular geographic units.

The simple aggregation analysis on the association between awareness of oral frailty and regional factors (see Table 2) indicated that awareness tended to decrease in certain municipalities. However, the multilevel analysis (see Table 3) did not reveal a significant association at the municipal level. The previous study reported regional differences in oral frailty awareness within a secondary medical care area, which comprises several municipalities within a single prefecture [14].

Several factors may explain the differences between this study’s findings and those of the previous study [14]. First, their study used logistic regression analysis on data from eight secondary medical care areas within one prefecture, whereas this study employed a multilevel analysis of 66 municipalities across Japan. Multilevel analysis offers the advantage of enabling a study to account for both individual- and municipal-level factors simultaneously. In addition, approximately 60% of the participants resided in urban or metropolitan areas in the previous study [14], whereas 60% of the municipalities were classified as rural in this study.

No significant associations were found between awareness of oral frailty and the implementation of municipal programs, such as leaflet distribution, resident lectures, or volunteer training. The only information available about these programs was whether they were implemented. Details such as the number of residents reached were not available, making it difficult to assess their effectiveness. A review of oral health programs in 791 municipalities suggested that the presence of dental hygienists and collaboration with related organizations are key factors in the effectiveness of such programs [33]. In a survey of 248 individuals aged 20 to 80 years [34], the most commonly reported sources of information about oral frailty were mass media (e.g., television and newspapers, 47.6%), the Internet (33.1%), and explanations provided by dentists and medical institutions (14.5%).

It is essential that municipalities evaluate the effectiveness of oral frailty intervention programs by assessing the outcomes of their own initiatives and systematically collecting and sharing information on successful practices.

In the sensitivity analysis, where the response “I have heard of the term” was categorized as indicating no awareness of oral frailty, the results were largely consistent with those of the main analysis. In particular, being male, having lower educational attainment, and lacking civic participation remained significantly associated with lack of awareness. This stricter classification was used to address the possibility that participants who are only “familiar” with the term may not fully understand its implications.

This study has several limitations. First, the response rate was 67.1%, making selection bias a possibility. Second, it remains unclear to what extent participants who reported being ‘familiar’ with the term ‘oral frailty’ actually understood its meaning. It is important to assess not only familiarity with the term but also related health behaviors. Future studies should evaluate both awareness and behavior to enhance the validity of the findings. Third, the sources through which participants became informed about oral frailty remain ambiguous. Although numerous preventive programs are currently being implemented, the scope and modalities of their dissemination have not been adequately documented. Consequently, it was not possible to assess the effectiveness of specific communication channels or interventions. Identifying credible sources of information and optimizing dissemination strategies are crucial for enhancing public awareness more effectively. Fourth, the study was unable to conduct analyses at the school district level due to the limited number of respondents; therefore, the analysis was conducted at the municipal level instead. Fifth, the measurement of oral and general health status was based on self-reports rather than clinical examinations.

## 5. Conclusions

This study clarified the regional differences in oral frailty awareness using cross-sectional data drawn from a questionnaire survey of older adults residing in municipalities across Japan. The study sought to identify the individual- and municipal-level factors associated with awareness, focusing on the presence of dental health ordinance and the implementation of oral health programs at the municipal level. The awareness of oral frailty across 66 municipalities ranged from 15.3% to 47.1%, representing a nearly threefold difference. Individual-level factors such as sex, presence of oral frailty, civic participation, and educational attainment were significantly associated with awareness, whereas no significant associations were observed with municipal-level factors. Enhancing awareness requires that more frequent civic participation among older adults be promoted in each municipality, particularly by fostering environments that support sustained engagement in social and recreational activities among men. In addition, implementing intervention programs that communicate to residents the importance of oral frailty prevention is critical for future public health efforts.

## Figures and Tables

**Table 1:** Characteristics of participants (*n* = 20,330).

Characteristics	Categories	Total	Awareness of Oral Frailty		
		*n*	*n*	%	*p* Value *
Sex	Men	9756	2450	25.1	<0.001
	Women	10,574	4434	41.9	
Age group (years)	65−69	4803	1700	35.4	<0.001
	70−74	6152	2157	35.1	
	75−79	4655	1605	34.5	
	80−84	3157	1027	32.5	
	≥85	1563	395	25.3	
Education attainment (years)	≤9	4025	829	20.6	<0.001
	10−12	8821	2975	33.7	
	≥13	7009	2941	42.0	
	Data missing	475	139	29.3	
Equivalent income (JPY 10,000)	0–199	8608	2571	29.9	<0.001
	200−399	7028	2683	38.2	
	≥400	2186	836	38.2	
	Data missing	2508	794	31.7	
Current illness	Yes	3792	1388	36.6	<0.001
	No	15,948	5301	33.2	
	Data missing	590	195	33.1	
Oral frailty	Yes	9514	2711	28.5	<0.001
	No	10,037	3951	39.4	
	Data missing	779	222	28.5	
Alcohol drinking status	Current drinker	8153	2551	31.3	<0.001
	Former drinker	2408	742	30.8	
	Never drinker	8993	3327	37.0	
	Data missing	776	264	34.0	
Smoking status	Current smoker	2021	475	23.5	<0.001
	Former smoker	6297	1720	27.3	
	Never smoker	11,673	4583	39.3	
	Data missing	339	106	31.3	
Frequency of going outside	≥5 times a week	12,306	4253	34.6	<0.001
	4 times a week	2750	1025	37.3	
	2−3 times a week	3616	1212	33.5	
	No	1351	294	21.8	
	Data missing	307	100	32.6	
Average of daily walking (min)	<30	4766	1338	28.1	<0.001
	30−59	7754	2809	36.2	
	≥60	7305	2575	35.2	
	Data missing	505	162	32.1	
Frequency of meeting friends	At least once/week	9201	3418	37.1	<0.001
	1−3 times/month	4579	1626	35.5	
	Rarely	6141	1694	27.6	
	Data missing	409	146	35.7	
Civic participation	Yes	7955	3439	43.2	<0.001
	No	11,782	3280	27.8	
	Data missing	593	165	27.8	
Reciprocity	Yes	19,863	6773	34.1	<0.001
	No	250	50	20.0	
	Data missing	217	61	28.1	
Social cohesion	Yes	17,987	6243	34.7	<0.001
	No	2159	595	27.6	
	Data missing	184	46	25.0	
Family structure	Living alone	3040	1129	37.1	<0.001
	Living with partner	9756	3391	34.8	
	Two households	3426	1101	32.1	
	Data missing	4108	1263	30.7	
Type of house	Owner-occupied house (detached)	16,332	5406	33.1	<0.001
	Owner-occupied house (apartment)	1574	712	45.2	
	Rented house	1798	580	32.3	
	Data missing	626	186	29.7	

* Chi-square test.

**Table 2:** Association between awareness of oral frailty and municipality-level variables (*n* = 66).

Variables		Categories	No. of Municipalities	Proportion of Individuals Aware of Oral Frailty (%)		
				Median	IQR	*p* Value *
Has a prefectural dental health ordinance been established?		Yes	58	30.8	25.7−36.3	0.398
		No	8	24.6	20.8−41.3	
Are oral frailty measures specified in prefectural dental health ordinances?		Yes	27	32.9	26.6−37.9	0.113
		No	39	29.7	22.8−35.8	
Has a municipal dental health ordinance been established?		Yes	15	33.3	25.5−40.3	0.371
		No	51	30.1	24.4−36.0	
Are oral frailty measures specified in municipal dental health ordinances?		Yes	5	32.9	25.0−36.4	0.925
		No	61	30.4	24.6−38.2	
Project related to oral frailty prevention	Distribution of leaflets	Yes	39	33.3	25.0−39.7	0.410
		No	11	30.3	24.4−32.8	
		Data missing	16	27.5	24.2−35.2	
	Lectures for citizens	Yes	36	32.5	25.8−39.9	0.274
		No	13	30.5	27.0−34.0	
		Data missing	17	27.0	23.5−34.4	
	Training of citizen volunteers	Yes	12	32.3	24.4−40.7	0.186
		No	35	32.8	25.8−39.7	
		Data missing	19	26.8	24.1−32.9	
Population density		Metropolitan	9	40.3	39.9−44.5	<0.001
		Urban	5	34.6	31.7−39.6	
		Semi-urban	40	30.1	25.0−33.8	
		Rural–agricultural	12	24.1	20.6−28.1	

IQR: interquartile range; * Mann–Whitney U test or Kruskal–Wallis test.

**Table 3:** Prevalence ratios for lack of awareness of oral frailty in individual- and municipal-level variables calculated using a multilevel Poisson regression model.

Variables	Categories	Model 1			Model 2			Model 3		
		PR	95% CI	*p* Value	PR	95% CI	*p* Value	PR	95% CI	*p* Value
**Fixed effects**										
Individual-level										
Sex	Men	1.11	1.08−1.13	<0.001	1.11	1.08−1.13	<0.001	1.10	1.07−1.13	<0.001
	Women	1.00	Reference		1.00	Reference		1.00	Reference	
Age group (years)	65−69	1.00	Reference		1.00	Reference		1.00	Reference	
	70−74	1.00	0.97−1.03	0.850	1.00	0.97−1.03	0.861	1.00	0.97−1.03	0.750
	75−79	1.01	0.98−1.04	0.605	1.01	0.98−1.04	0.642	0.99	0.96−1.03	0.503
	80−84	1.02	0.99−1.06	0.249	1.02	0.99−1.06	0.249	0.99	0.96−1.02	0.747
	≥85	1.06	1.02−1.11	0.005	1.06	1.02−1.11	0.008	1.01	0.96−1.02	0.575
Educational attainment (years)	≤9	1.13	1.10−1.17	<0.001				1.10	1.06−1.14	<0.001
	10−12	1.05	1.02−1.08	<0.001				1.04	1.02−1.07	0.001
	≥13	1.00	Reference					1.00	Reference	
	Data missing	1.08	1.00−1.16	0.040				1.05	0.98−1.13	0.157
Equivalent income (JPY 10,000)	0−199	1.05	1.01−1.09	0.013				1.03	0.99−1.07	0.196
	200−399	1.00	0.96−1.04	0.987				1.00	0.96−1.04	0.891
	≥400	1.00	Reference					1.00	Reference	
	Data missing	1.04	0.99−1.08	0.124				1.02	0.97−1.07	0.481
Current illness	Yes	1.00	Reference					1.00	Reference	
	No	1.02	0.99−1.05	0.162				1.00	0.98−1.03	0.740
	Data missing	1.02	0.96−1.09	0.528				1.02	0.95−1.09	0.674
Oral frailty	Yes	1.07	1.04−1.09	<0.001				1.04	1.01−1.06	0.005
	No	1.00	Reference					1.00	Reference	
	Data missing	1.07	1.01−1.13	0.025				1.05	0.98−1.12	0.152
Alcohol drinking status	Current drinker	1.04	1.01−1.06	0.002				1.00	0.97−1.02	0.756
	Former drinker	1.04	1.00−1.08	0.029				0.99	0.95−1.02	0.454
	Never drinker	1.00	Reference					1.00	Reference	
	Data missing	1.02	0.96−1.08	0.535				0.99	0.92−1.06	0.705
Smoking status	Current smoker	1.10	1.06−1.14	<0.001				1.02	0.98−1.07	0.280
	Former smoker	1.08	1.05−1.10	<0.001				1.01	0.98−1.05	0.391
	Never smoker	1.00	Reference					1.00	Reference	
	Data missing	1.05	0.97−1.14	0.256				1.03	0.89−1.19	0.708
Frequency of going outside	≥5 times a week	1.00	Reference					1.00	Reference	
	4 times a week	0.99	0.95−1.02	0.374				0.99	0.95−1.02	0.389
	2−3 times a week	1.01	0.98−1.04	0.602				0.99	0.96−1.02	0.522
	No	1.08	1.03−1.12	0.001				1.02	0.98−1.07	0.319
	Data missing	1.01	0.93−1.11	0.761				0.97	0.83−1.13	0.660
Average of daily walking (min)	<30	1.04	1.01−1.07	0.004				1.02	0.99−1.05	0.129
	30−59	1.00	0.97−1.02	0.748				1.00	0.98−1.03	0.993
	≥60	1.00	Reference					1.00	Reference	
	Data missing	1.02	0.95−1.09	0.613				1.00	0.91−1.10	0.975
Frequency of meeting friends	At least once/week	1.00	Reference					1.00	Reference	
	1−3 times/month	1.01	0.99−1.04	0.390				1.00	0.97−1.03	0.924
	Rarely	1.06	1.04−1.09	<0.001				1.01	0.99−1.04	0.355
	Data missing	1.01	0.94−1.09	0.804				0.96	0.86−1.06	0.416
Civic participation	Yes	1.00	Reference					1.00	Reference	
	No	1.10	1.07−1.12	<0.001				1.06	1.03−1.08	<0.001
	Data missing	1.09	1.03−1.17	0.006				1.05	0.98−1.13	0.185
Reciprocity	Yes	1.00	Reference					1.00	Reference	
	No	1.09	0.99−1.19	0.079				1.02	0.93−1.12	0.699
	Data missing	1.04	0.94−1.15	0.495				1.04	0.90−1.20	0.592
Social cohesion	Yes	1.00	Reference					1.00	Reference	
	No	1.04	1.01−1.08	0.018				1.02	0.98−1.06	0.286
	Data missing	1.06	0.95−1.18	0.334				1.02	0.91−1.14	0.744
Family structure	Living alone	1.00	Reference					1.00	Reference	
	Living with partner	1.02	0.98−1.05	0.376				1.01	0.98−1.04	0.616
	Two households	1.03	0.99−1.07	0.140				1.03	0.99−1.07	0.180
	Data missing	1.04	1.00−1.07	0.059				1.03	0.99−1.07	0.165
Type of house	Owner-occupied house (detached)	1.06	1.02−1.11	0.005				1.03	0.99−1.08	0.179
	Owner-occupied house (apartment)	1.00	Reference					1.00	Reference	
	Rented house	1.08	1.02−1.14	0.008				1.03	0.97−1.09	0.300
	Data missing	1.09	1.01−1.17	0.027				1.03	0.96−1.12	0.375
Municipal-level										
Civic participation (mean)		0.68	0.57−0.80	<0.001				0.87	0.70−1.07	0.190
Reciprocity (mean)		0.96	0.14−6.65	0.965				2.16	0.32−14.56	0.430
Social cohesion (mean)		0.61	0.38−0.98	0.041				0.82	0.52−1.29	0.381
Include oral frailty in a prefectural ordinance	Yes	1.00	Reference					1.00	Reference	
	No	1.01	0.99−1.04	0.353				0.99	0.96−1.01	0.286
Training of citizen volunteers	Yes	1.00	Reference					1.00	Reference	
	No	1.01	0.98−1.04	0.526				1.02	0.99−1.04	0.283
	Data missing	1.04	1.00−1.08	0.061				1.02	0.98−1.06	0.312
Population density	Metropolitan	1.00	Reference					1.00	Reference	
	Urban	1.04	0.99−1.09	0.118				1.02	0.97−1.07	0.445
	Semi-urban	1.07	1.04−1.09	<0.001				1.02	0.99−1.06	0.129
	Rural–agricultural	1.10	1.06−1.15	<0.001				1.03	0.98−1.09	0.262
**Random effects**										
Municipal-level variance (SE)		0.0008 (0.0005)			0.0009 (0.0005)			0.0000 (0.0000)		

PR: prevalence ratio; CI: confidence interval; SE: standard error. Model 1: Each individual- and municipal-level variable was added separately to the two-level multilevel Poisson regression model. The value of the random effects was that for the null model. Model 2: Sex and age groups were added simultaneously to the two-level multilevel Poisson regression model. Model 3: All individual- and municipal-level variables were added simultaneously to the two-level multilevel Poisson regression model.

**Table 4:** Sensitivity analyses showing the prevalence of a conservative definition of awareness (‘very familiar’ only) and prevalence ratios for lack of awareness (‘have heard of the term’ and ‘not familiar’) using the multilevel Poisson regression model.

Variables	Categories	Total	Awareness of Oral Frailty		Multilevel Poisson Regression *		
		*n*	*n*	%	PR	95%CI	*p* Value
Sex	Men	9756	546	5.6	1.03	1.01−1.06	0.003
	Women	10,574	1071	10.1	1.00	Reference	
Age group (years)	65−69	803	354	7.4	1.00	Reference	
	70−74	6152	480	7.8	0.99	0.97−1.02	0.506
	75−79	4655	393	8.4	0.98	0.96−1.01	0.196
	80−84	3157	282	8.9	0.98	0.95−1.01	0.110
	≥85	1563	108	6.9	0.98	0.95−1.02	0.343
Educational attainment (years)	≤9	4025	158	3.9	1.04	1.02−1.07	0.001
	10−12	8821	625	7.1	1.03	1.01−1.05	0.010
	≥13	7009	797	11.4	1.00	Reference	
	Data missing	475	37	7.8	1.01	0.96−1.07	0.612
Equivalent income (JPY 10,000)	0−199	8608	529	6.1	1.03	1.01−1.07	0.022
	200−399	7028	648	9.2	1.02	0.99−1.05	0.160
	≥400	2186	268	12.3	1.00	Reference	
	Data missing	2508	172	6.9	1.03	1.00−1.07	0.093
Current illness	Yes	3792	340	9.0	1.00	Reference	
	No	15,948	1238	7.8	1.00	0.98−1.03	0.739
	Data missing	590	39	6.6	1.01	0.96−1.07	0.601
Oral frailty	Yes	9514	609	6.4	1.01	1.00−1.03	0.168
	No	10,037	954	9.5	1.00	Reference	
	Data missing	779	54	6.9	1.02	0.97−1.07	0.533
Alcohol drinking status	Current drinker	8153	596	7.3	1.00	0.98−1.02	0.965
	Former drinker	2408	165	6.9	1.00	0.97−1.03	0.866
	Never drinker	8993	797	8.9	1.00	Reference	
	Data missing	776	59	7.6	1.01	0.95−1.06	0.844
Smoking status	Current smoker	2021	87	4.3	1.01	0.98−1.04	0.707
	Former smoker	6297	396	6.3	1.00	0.98−1.03	0.920
	Never smoker	11,673	1107	9.5	1.00	Reference	
	Data missing	339	27	8.0	1.01	0.91−1.13	0.823
Frequency of going outside	≥5 times a week	12,306	1050	8.5	1.00	Reference	
	4 times a week	2750	234	8.5	1.00	0.98−1.03	0.955
	2−3 times a week	3616	240	6.6	1.01	0.98−1.03	0.632
	No	1351	66	4.9	1.00	0.97−1.04	0.806
	Data missing	307	27	8.8	0.98	0.87−1.11	0.751
Average of daily walking (min)	<30	4766	300	6.3	1.01	0.99−1.03	0.503
	30−59	7754	632	8.2	1.01	0.99−1.03	0.565
	≥60	7305	641	8.8	1.00	Reference	
	Data missing	505	44	8.7	0.99	0.92−1.07	0.800
Frequency of meeting friends	At least once/week	9201	894	9.7	1.00	Reference	
	1−3 times/month	4579	343	7.5	1.01	0.99−1.03	0.404
	Rarely	6141	349	5.7	1.01	0.99−1.03	0.453
	Data missing	409	31	7.6	1.00	0.93−1.09	0.914
Civic participation	Yes	7955	934	11.7	1.00	Reference	
	No	11,782	643	5.5	1.03	1.01−1.05	0.004
	Data missing	593	40	6.7	1.02	0.96−1.08	0.560
Reciprocity	Yes	19,863	1592	8.0	1.00	Reference	
	No	250	11	4.4	1.00	0.92−1.07	0.911
	Data missing	217	14	6.5	1.00	0.90−1.12	0.973
Social cohesion	Yes	17,987	1491	8.3	1.00	Reference	
	No	2159	118	5.5	1.01	0.98−1.03	0.660
	Data missing	184	8	4.3	1.01	0.93−1.11	0.793
Family structure	Living alone	3040	262	8.6	1.00	Reference	
	Living with partner	9756	831	8.5	1.00	0.97−1.03	0.934
	Two households	3426	254	7.4	1.01	0.98−1.04	0.528
	Data missing	4108	270	6.6	1.01	0.98−1.04	0.452
Type of house	Owner-occupied house (detached)	16,332	1269	7.8	1.01	0.98−1.05	0.405
	Owner-occupied house (apartment)	1574	189	12.0	1.00	Reference	
	Rented house	1798	117	6.5	1.02	0.98-1.06	0.435
	Data missing	626	42	6.7	1.01	0.96-1.08	0.629
Municipal-level civic participation (mean)					0.95	0.80−1.11	0.503
Municipal-level reciprocity (mean)					1.88	0.43−8.19	0.400
Municipal-level social cohesion (mean)					0.85	0.60−1.04	0.555
Include oral frailty in a prefectural ordinance	Yes				1.00	Reference	
	No				0.99	0.97-1.01	0.454
Training of citizen volunteers	Yes				1.00	Reference	
	No				1.01	0.99−1.03	0.354
	Data missing				1.01	0.98−1.04	0.555
Population density	Metropolitan				1.00	Reference	
	Urban				1.01	0.97−1.05	0.722
	Semi-urban				1.00	0.98−1.03	0.731
	Rural–agricultural				1.01	0.97−1.05	0.687

PR: prevalence ratio; CI: confidence interval. * Prevalence ratios for lack of awareness of oral frailty in individual- and municipal-level variables were calculated using a two-level multilevel Poisson regression model. Municipal-level variances (standard errors) of the null model and the full model were 0.0000 (0.0000) and 0.0000 (0.0000), respectively.

## Data Availability

All data used are from the Japan Gerontological Evaluation Study (JAGES) and are not third-party data. All enquiries are to be addressed to the JAGES data management committee via e-mail: dataadmin.ml@jages.net. All JAGES datasets have ethical or legal restrictions for public deposition due to the inclusion of sensitive information from the human participants. Following the regulation of local governments which cooperated on our survey, the JAGES data management committee has imposed restrictions upon the data.

## Abbreviations

The following abbreviations are used in this manuscript:

CI	Confidence interval
IQR	Interquartile range
JAGES	Japan Gerontological Evaluation Study
PR	Prevalence ratio
SE	Standard error
